# Time-series clustering analysis reveals distinct patterns of cytomegalovirus viremia in critically ill adults

**DOI:** 10.1186/s40635-026-00866-9

**Published:** 2026-02-12

**Authors:** Jan-Hendrik Naendrup, Oliver Martin Hilbers, Henning Gruell, Lisa Altenrath, Jan-Michel Heger, Dennis Alexander Eichenauer, Boris Böll, Matthias Kochanek, Alexander Shimabukuro-Vornhagen, Jorge Garcia Borrega

**Affiliations:** 1 Department I of Internal Medicine, Center for Integrated Oncology Aachen Bonn Cologne Düsseldorf (CIO ABCD), Faculty of Medicine and University Hospital Cologne, University of Cologne, Cologne, Germany; 2 Department of Surgery, St. Elisabeth Hospital, Cologne, Germany; 3 Institute of Virology, Faculty of Medicine and University Hospital Cologne, University of Cologne, Cologne, Germany

**Keywords:** CMV reactivation, CMV disease, Viral load, Treatment threshold, Ganciclovir

## Abstract

**Background:**

Critically ill patients are at increased risk for cytomegalovirus (CMV) reactivation, which is associated with poorer clinical outcomes. However, little is known about the longitudinal viremia trajectories in this population.

**Methods:**

This retrospective single-center study was conducted in a medical ICU and included patients with CMV viremia ≥ 1000 International Units CMV–DNA per milliliter whole blood (IU/mL) treated between March 2014 and April 2021. Time-series clustering was applied to identify subgroups of patients with similar longitudinal viremia trajectories.

**Results:**

82 patients were included in the final analysis. Time-series clustering identified three distinct clusters: (1) patients with initial high viremia (median 46,700 IU/mL), 94% receiving treatment and showing subsequent steep reduction of viremia; (2) patients with moderate viremia (median 2720 IU/mL) and subsequent increase in viral load, treated in 52%; and (3) patients with moderate viremia (median 3120 IU/mL), 63% receiving treatment and showing stable viral load in follow-up measurements. No significant differences were identified between the clusters with respect to patient characteristics, including underlying immunosuppression. With respect to disease severity, the Acute Physiology and Chronic Health Evaluation II (APACHE-II) score was highest in cluster 3 and among patients without follow-up CMV–DNA measurements (*P* = 0.029), while the Sequential Organ Failure Assessment (SOFA) score demonstrated a similar directional trend without reaching statistical significance. Survival differed significantly between the clusters in the Kaplan–Meier estimate (*p* = 0.008); however, absolute 1-year survival was low across all clusters (cluster 1: 0%, cluster 2: 33%, cluster 3: 32%, patients without follow-up CMV measurement: 14%; *p* = 0.062). Probable CMV pneumonia with respiratory symptoms and CMV–DNA detection in bronchoalveolar lavage fluid was the most common disease manifestation (cluster 1: 35%; cluster 2: 28%; cluster 3: 7.5%; patients without follow-up CMV measurement: 23%; *p* = 0.040).

**Conclusions:**

In this hypothesis-generating study, time-series clustering analysis identified three subgroups with distinct longitudinal viremia trajectories which significantly differed in viral load, treatment decisions and survival over time. The diagnostic and therapeutic relevance of longitudinal CMV viremia trajectories and the optimal CMV–DNA threshold for treatment initiation in ICU patients remain undefined and might differ from other cohorts.

**Supplementary Information:**

The online version contains supplementary material available at 10.1186/s40635-026-00866-9.

## Background

Cytomegalovirus (CMV) is a ubiquitous herpesvirus with high seroprevalence rates in adults [1, 2]. Following primary infection, the virus maintains latency and may reactivate under conditions of systemic inflammation or diminished immune function [3–5]. As a result of immune dysregulation, critically ill patients in the intensive care unit (ICU) are at increased risk for CMV reactivation [4, 6, 7]. Multiple studies have shown that CMV reactivation and high CMV viral load are associated with worse clinical outcomes in ICU patients [7–9]. However, whether CMV reactivation is a contributing factor or merely a reflection of inflammation and disease severity is currently unclear [9–11].

Thresholds for the initiation of antiviral therapy in the presence of CMV reactivation without disease manifestation vary among patient populations and institutions. In immunocompromised patients, such as following solid organ transplant (SOT) or allogeneic hematopoietic stem-cell transplantation (HSCT), CMV viral load monitoring with pre-emptive treatment to prevent CMV disease is well-established [12, 13]. In non-immunosuppressed ICU patients without CMV disease, the benefit of antiviral treatment remains controversial as neither “pre-emptive” treatment in patients with CMV–DNA viremia nor “prophylactic” treatment in CMV–IgG-seropositive individuals resulted in improved clinical outcomes [14, 15].

Despite the ongoing debates, little is known about the longitudinal viremia trajectories in critically ill patients and real-world data on CMV viremia evolvement with and without antiviral intervention is limited [15]. This study aimed to retrospectively assess the prognostic significance of different CMV viral trajectories and to elucidate the influence of antiviral therapy on these virological patterns.

## Methods

We performed a retrospective single-center study in the medical ICU of a tertiary university hospital (March 2014–April 2021). Eligible patients had CMV viremia with a viral load of ≥ 1000 International Units per milliliter (IU/mL) in whole blood detected by quantitative polymerase chain reaction (qPCR) during the ICU stay. qPCR analysis was performed on a LightCycler^®^ 480 Instrument (Roche Diagnostics, Basel, Switzerland) according to the manufacturer’s instructions. Indications for CMV–DNA measurements in our ICU included: (1) routine surveillance with biweekly measurements in severely immunocompromised patients following solid organ transplantation (SOT) or allogeneic hematopoietic stem-cell transplantation (HSCT); (2) targeted testing in patients with acquired immunodeficiency syndrome (AIDS) due to confirmed human immunodeficiency virus (HIV) infection, as well as in patients with suspected hemophagocytic lymphohistiocytosis (HLH) based on the HScore; and (3) individual diagnostic testing in non-immunocompromised patients presenting with persistent fever or ongoing laboratory evidence of inflammation without an identifiable infectious focus or causative pathogen despite comprehensive microbiological and virological diagnostics and appropriate medical imaging. In the latter group, the timing and indication of CMV–DNA measurement were non-standardized and physician-driven.

To allow for the analysis of longitudinal viremia trajectories, only patients with at least one subsequent CMV–DNA measurement during the ICU stay were included. The patient population consisted of both patients who had CMV-directed antiviral treatment and patients with subsequent CMV–DNA monitoring without treatment. To analyze the treatment effect on longitudinal viremia trajectories, only patients in whom CMV-directed antiviral therapy was started within ± 3 days of CMV viremia were included. The ± 3 day window was chosen a priori to reflect local biweekly surveillance practices in high-risk patients and to ensure temporal proximity between viremia detection and treatment initiation. Demographics, comorbidities, immunosuppression status, laboratory data, ICU interventions, antiviral therapy, and outcomes were extracted from medical records. Immunosuppression was defined according to the criteria outlined in the UK Health Security Agency’s Green Book. Viremia was assessed longitudinally based on serial CMV–PCR results, with follow-up measurements on the general ward being excluded. Proven and probable CMV disease were defined according to CMV Forum consensus criteria [16]. In brief, proven end-organ disease requires compatible clinical manifestations and direct demonstration of CMV in the affected tissue, e.g., by histopathology, virus isolation or rapid culture. Possible end-organ disease may be inferred from CMV–DNA levels in organ tissue, e.g., in case of probable CMV pneumonia the detection of CMV–DNA in the bronchoalveolar lavage. Antiviral therapy was defined as administration of ganciclovir, valganciclovir, or foscarnet in a therapeutic dosage.

### Statistical analysis

All analyses were performed using R version 4.5.0 (ZB: R: A Language and Environment for Statistical Computing. R Foundation for Statistical Computing, Vienna, Austria. https://www.R-project.org/.) and RStudio (R version 2025.5.0.496; RStudio Inc., Boston, MA, USA). Continuous variables were presented as medians with interquartile ranges (IQR; Q1, Q3), while categorical variables were summarized as absolute numbers and percentages [n (%)]. Comparisons between groups were conducted using the Wilcoxon rank-sum test for continuous variables and Pearson’s chi-squared test or Fisher’s exact test for categorical variables, as appropriate. Survival was analyzed with Kaplan–Meier curves and log-rank tests. Significance was set at *p* < 0.05.

Viral load values were log- and z-transformed for comparability across patients. Time-series clustering was performed using the “tsclust” in R package to identify subgroups with similar longitudinal viremia dynamics. Clustering was performed using a partitional approach in which patients were grouped based on the similarity of their longitudinal viremia trajectories. Similarity between trajectories was quantified using dynamic time warping (DTW) distance, which allows for temporal shifts in the patterns (e.g., peaks occurring at slightly different times). Each cluster was represented by a centroid computed with DTW barycenter averaging (DBA), providing a representative trajectory that captures the overall temporal pattern within the cluster. This approach allows comparison of patients with heterogeneous sampling frequency and timing, which is common in retrospective ICU data sets. The optimal number of clusters was initially explored using internal cluster validity indices, specifically average silhouette width and the Davies–Bouldin index.

## Results

Of 140 ICU patients with CMV viremia ≥ 1000 IU/mL, 27 were excluded (7 due to incomplete records, 20 due to antiviral therapy initiated outside the predefined ± 3 day window). Eighty-two patients remained for the clustering analysis, while 31 patients had no subsequent CMV–DNA measurement and are presented as “no cluster” group.

The average silhouette width and Davies–Bouldin index most frequently favored a two-cluster solution, reflecting dominant separation by overall viral burden; however, across different initializations and cluster solutions, these indices did not consistently identify a unique optimal number of clusters and occasionally supported a three-cluster structure. Examination of the longitudinal trajectories revealed that patients within the moderate viremia range consistently exhibited two distinct and reproducible temporal patterns, i.e., progressive viral amplification vs. stable viral persistence, that were merged in a two-cluster solution. Given the exploratory nature of the study and the presence of two reproducible and clinically distinct kinetic patterns within the moderate viremia range, a three-cluster solution was selected despite less support from internal validity indices.

**Fig. 1:**
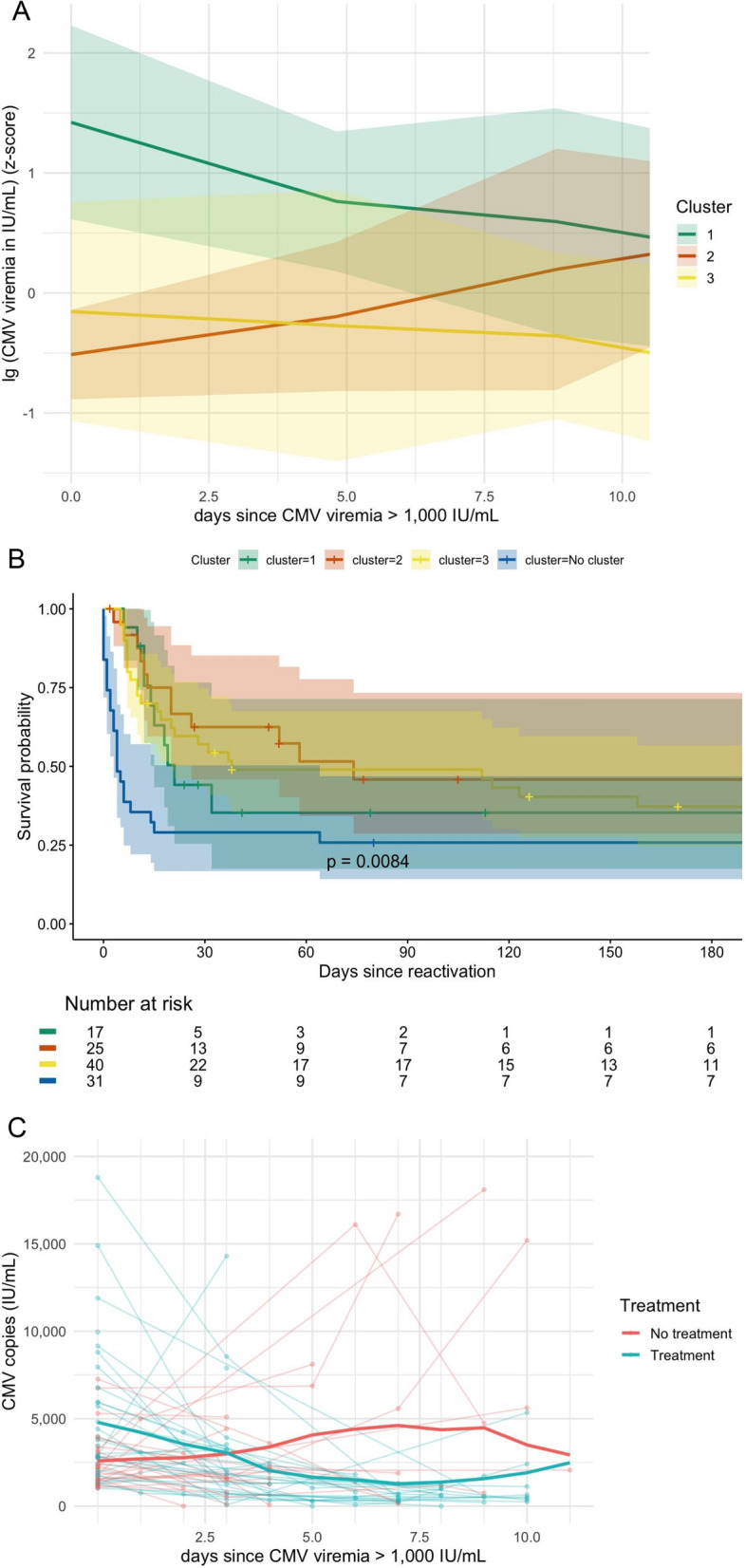
**A** Mean and standard deviation of clustered longitudinal viral load trajectories of patients with CMV viremia $\ge$ 1000 IU/mL and at least one follow-up CMV–DNA measurement on the intensive care unit (log- and z-transformed) (Not the entire patient population is shown, only patients with at least one follow-up CMV–DNA measurement.); **B** Kaplan–Meier analysis of the identified clusters of different viremia trajectories and the group without at least one follow-up CMV–DNA measurement (“no cluster”); **C** viremia trajectories of patients with and without antiviral treatment in the moderate viremia subgroups of clusters 2 and 3

The identified clusters are illustrated in Fig. 1a. Clustered longitudinal viremia trajectories of individual patients are illustrated in supplement Fig. 1. In summary, cluster 1 comprised a patient population with initially high viral load, mostly exceeding 10,000 IU/mL that subsequently showed viral load reduction. Cluster 2 and cluster 3 comprised patients with moderate viremia, usually between 1000 and 10,000 IU/mL. Within this subgroup, patients in cluster 2 illustrated a subsequent increase of viremia, while cluster 3 showed a relatively stable viral load in follow-up measurements.

The patient baseline characteristics and outcomes of the different clusters are listed in Table 1 and supplement Table 1. Significant differences between the different clusters were identified regarding initial viral load (*p* < 0.001) and CMV-directed antiviral treatment, with the majority of patients in the high viremia cluster 1 receiving antiviral treatment (94%) and approximately half of the patients receiving treatment in the moderate viremia cluster 2 and 3 (52% in cluster 2; 63% in cluster 3; 52% in the “no cluster” group; *p* = 0.019). With respect to disease severity, the Acute Physiology and Chronic Health Evaluation II (APACHE-II) score was significantly higher in cluster 3 with a median of 22 [quartile1–quartile3 (16–26)] and in the “no cluster” group with a median of 21(16–27) compared to cluster 1 and 2 with a median of 18 (*p* = 0.029). In contrast, the Sequential Organ Failure Assessment (SOFA) score demonstrated a similar trend without reaching statistical significance. All patients receiving antiviral therapy were treated with ganciclovir. No significant differences between the clusters were detected regarding the overall prevalence of proven or probable CMV disease. Probable CMV pneumonia with respiratory symptoms and detection of CMV–DNA in the bronchoalveolar lavage was the most frequent manifestation of CMV disease and was significantly more prevalent in cluster 1 [cluster 1: 6 (35%); cluster 2: 7 (28%); cluster 3: 3 (7.5%); “no cluster” group: 23%; *p* = 0.040]. Proven or probable gastrointestinal disease was the second most common CMV disease manifestation [cluster 1: 1 (5.9%); cluster 2: 2 (8.0%); cluster 3: 6 (15%); “no cluster” group: 13%; *p* = 0.8]. The Kaplan–Meier analysis of the different clusters is illustrated in Fig. 1b, showing significant survival differences over time between the clusters (*p* = 0.008) with the “no cluster” group having the lowest survival probability. Overall, 1-year survival rates were low for all three clusters (0%, 33%, and 32%, respectively).

**Table 1:** Patient characteristics of patients with cytomegalovirus (CMV) viremia $\ge$1,000 IU/mL on the intensive care unit (ICU) with and without CMV-directed antiviral treatment (treatment initiation $\pm$3 days of viremia), clustered by longitudinal viral load trajectories

Patient characteristics	*N*	cluster 1 *N* = 17^*1*^	cluster 2 *N* = 25^*1*^	cluster 3 *N* = 40^*1*^	No cluster *N* = 31^*1*^	*p* value^*2*^
Age	113	64.0 (40.0, 69.0)	57.0 (47.0, 67.0)	61.0 (47.5, 66.0)	59.0 (52.0, 67.0)	>0.9
Sex	113					0.2
Female		4 (24%)	4 (16%)	11 (28%)	13 (42%)	
Male		13 (76%)	21 (84%)	29 (73%)	18 (58%)	
CCI	113	5.0 (4.0, 6.0)	5.0 (2.0, 7.0)	4.0 (3.0, 6.0)	4.0 (2.0, 6.0)	0.6
SOFA score*	113	5.0 (4.0, 7.0)	5.0 (4.0, 9.0)	7.5 (5.0, 12.0)	8.0 (4.0, 11.0)	0.12
APACHE-II score*	112	18.0 (14.0, 20.0)	18.0 (13.0, 22.0)	22.0 (16.0, 26.0)	21.0 (16.0, 27.0)	0.029
Immunosuppression	113	15 (88%)	18 (72%)	28 (70%)	24 (77%)	0.5
Solid organ transplant	113	1 (5.9%)	3 (12%)	4 (10%)	1 (3.2%)	0.6
Allogeneic HSCT	113	1 (5.9%)	5 (20%)	12 (30%)	6 (19%)	0.2
HIV	113	5 (29%)	4 (16%)	4 (10%)	4 (13%)	0.3
CMV copies (IU/mL) *	113	46,700.0 (19,400.0, 117,000.0)	2,720.0 (1,560.0, 3,790.0)	3,120.0 (1,808.0, 8,985.0)	2,880.0 (1,670.0, 9,980.0)	<0.001
Proven/probable CMV disease	113	9 (53%)	10 (40%)	11 (28%)	13 (42%)	0.3
Treatment						
CMV-directed treatment	113	16 (94%)	13 (52%)	25 (63%)	16 (52%)	0.019
Vasopressors	113	14 (82%)	18 (72%)	28 (70%)	21 (68%)	0.8
Mechanical ventilation	113	11 (65%)	16 (64%)	27 (68%)	19 (61%)	>0.9
RRT	113	4 (24%)	10 (40%)	11 (28%)	13 (42%)	0.4
ECMO	113	0 (0%)	1 (4.0%)	3 (7.5%)	4 (13%)	0.5
CPR	113	4 (24%)	3 (12%)	10 (25%)	7 (23%)	0.6
Outcome						
ICU length of stay	113	19.0 (15.0, 22.0)	30.0 (13.0, 34.0)	21.5 (8.0, 51.0)	9.0 (5.0, 22.0)	0.010
30-day survival	113	8 (47%)	20 (80%)	25 (63%)	8 (26%)	<0.001
ICU survival	113	8 (47%)	16 (64%)	23 (58%)	9 (29%)	0.039
Hospital survival	113	7 (41%)	14 (56%)	20 (50%)	6 (19%)	0.022
1-year survival	91	0 (0%)	6 (33%)	11 (32%)	4 (14%)	0.062

The “no cluster” group presents patients without at least one follow-up CMV–DNA measurement, who, therefore, could not be clustered

^*1*^ Median (Q1, Q3); *n* (%)

^*2*^ Kruskal–Wallis rank-sum test; Fisher’s exact test; Pearson’s chi-squared test

CCI: Charlson Comorbidity Index; SOFA score: Sequential Organ Failure Assessment score; APACHE-II score: Acute Physiology and Chronic Health Evaluation II score; HSCT: hematopoietic stem-cell transplantation; HIV human immunodeficiency virus; RRT: renal replacement therapy; ECMO: extracorporeal membrane oxygenation; CPR: cardiopulmonary resuscitation

The longitudinal viremia trajectories of patients in cluster 2 and 3, with approximately equal distribution of antiviral treatment vs. monitoring without antiviral treatment are illustrated in Fig. 1c. The comparison of patients with and without antiviral treatment from all available data, including patients without longitudinal CMV viral load assessments, is listed in supplement Tables 1 and 2, a Kaplan–Meier analysis is shown in supplement Fig. 2. In addition, in this collective group, including patients without follow-up viral load (*n* = 113), no significant differences regarding clinical outcome parameters were observed between patients with and without antiviral treatment.

## Discussion

CMV viremia is a frequent observation with uncertain relevance in critically ill adults, yet its prognostic significance and optimal management remain uncertain. This hypothesis-generating study used time-series clustering to identify three distinct CMV viremia trajectories in ICU patients, differing significantly in terms of baseline viral load, treatment decision, and survival over time in the Kaplan–Meier estimate. However, the observations also highlight the ambiguity surrounding current treatment strategies regarding viral thresholds and indications for initiating treatment in ICU patients without CMV disease.

Primarily, one subgroup distinguished from the others, characterized by a high initial viral load and rapid dynamics in subsequent viral reduction with almost all patients receiving antiviral treatment (Fig. 1a). Although no significant differences compared to the other subgroups were observed in the fraction of immunosuppressed patients or in the subgroups of post-transplant and HIV patients, details regarding the intensity of immunosuppression or the specific immunosuppressive regimens were not provided. Based on data from non-ICU transplant populations, it is plausible that both the degree of immunosuppression and the type of immunosuppressive therapy likely also play a critical role in CMV reactivation in critically ill ICU patients [17–19].

Interestingly, the severity of illness, represented by APACHE-II score, was higher in the cluster with a relatively stable viral load in follow-up measurements (cluster 3) as well as in the patients without follow-up measurements and not in the high viremia cluster (cluster 1). In this regard, it should be noted that while disease severity is associated with the prevalence of CMV reactivation, as recently underlined by identifying the APACHE-II scores as independent risk factor for CMV reactivation [20], a clear correlation between disease severity and CMV viral load has not been demonstrated in ICU patients. Consequently, CMV reactivation, rather than viral load has been proposed as a surrogate marker of critical illness severity [21].

Yet at the same time, data from HIV and transplant patients indicated that a high viral load is associated with a poor outcome in terms of mortality and complications [9, 22–25]. In addition, in the present data, the cluster with high viremia is associated with lower survival over time in the Kaplan–Meier estimate and only patients without follow-up measurements did worse.

Against the backdrop of the associated mortality and along with a more than tenfold higher median viremia compared to the other clusters, the restraints to initiate antiviral therapy in cluster 1 appears to be low among treating physicians. 94% of patients in the rapidly declining high viremia cluster received treatment, while physicians decided to initiate treatment in the moderate viremia group only in about half of the patients. Initiation of pre-emptive therapy in this group may have been driven by data from immunocompromised patients showing antiviral treatment efficacy in preventing tissue-invasive disease [26–28]. In case of probable CMV pneumonia, which was the most prevalent manifestation of CMV disease in the present analysis, it has recently been shown that involvement of the lower respiratory tract is associated with poor outcomes in critically ill patients [8]. In the high viremia cluster, more than a third of patients showed probable CMV pneumonia, defined by respiratory symptoms and detection of CMV–DNA in the bronchoalveolar lavage. It should be acknowledged that the diagnostic criteria for CMV pneumonia were originally established for the use in immunocompromised patient populations, in whom the pre-test probability of clinically relevant infection is substantially increased compared to the general ICU population. In the most recent revision of these criteria, the threshold values for CMV–DNA copy numbers in bronchoalveolar lavage fluid were refined [29]. As quantitative data on CMV–DNA levels were not available for the present analysis, the possibility that in some cases the detected viral DNA reflects asymptomatic viral shedding rather than active disease cannot be excluded.

Regarding antiviral treatment, the data in ICU patients is rather austere. A recent randomized clinical trial by Papazian and colleges in mechanically ventilated ICU patients that exhibited CMV reactivation > 500 IU/mL was prematurely terminated based on the interim analysis by the data safety monitoring board due to the high number of patients necessary to show differences. Although the analysis of 76 randomized patients (39 ganciclovir, 37 placebo) showed no improvement in clinical outcome when treating pre-emptively with ganciclovir, the lack of statistical power precludes any recommendation in favor of or against the pre-emptive use of ganciclovir [14]. In addition, the use of ganciclovir as a prophylactic agent among CMV-seropositive ICU patients did not reduce the biological primary end point of change in plasma interleukin 6 (IL-6) levels between baseline and day 14 in a placebo-controlled, randomized clinical trial by Limaye and colleges. As a secondary outcome, CMV reactivation in plasma was significantly lower in the ganciclovir group, while other secondary outcomes showed no significant differences. Importantly, the study was also not powered to identify differences in the secondary clinical outcome. [15]. Although a reduction of the viral load was observed in the high viremia subgroup, the study design does not allow attribution of this decline to antiviral therapy. In addition, in immunocompromised non-ICU patients the appropriate threshold to initiate pre-emptive treatment to prevent CMV disease is debatable, and recommendations vary [30–32]. The data available for ICU patients is even more limited, and the lack of thresholds for pre-emptive treatment is reflected by the finding that only half of the patients received antiviral treatment in the moderate viremia subgroup, mostly ranging between 1000 and 10,000 IU/mL. Descriptively, the longitudinal course of CMV viremia appears to differ in the first few days depending on the treatment; however, the viral loads converge substantially by day 10 following the first CMV viremia $\ge$ 1000 IU/mL on ICU (Fig. 1c). Those findings seem to underline the present body of evidence that antiviral treatment in ICU patients without disease manifestation and moderate viral load need to be carefully weighed against potentially side effects, such as bone marrow suppression or nephropathy. It should also be considered that targeted antiviral treatment in these cases might primarily lower viral load measurements without substantially impacting the underlying pathophysiological processes in these critically ill patients.

This analysis has several important limitations. First, the retrospective study design, the limited sample size, and the absence of histological or autopsy specimens restrict causal inference and preclude conclusions regarding CMV viremia in ICU patients. In particular, the clinical management decisions regarding CMV follow-up measurements and treatment were not standardized and were subject to physician discretion, introducing significant selection bias. Importantly, clustering strategy was conditioned on observed longitudinal CMV kinetics, which are themselves influenced by clinical decision-making, thereby increasing the risk of informative censoring and treatment-induced trajectory. As CMV kinetics are influenced by clinical decisions—particularly the timing of antiviral therapy initiation—these trajectories may not accurately reflect the natural history of CMV reactivation. The lack of data on CMV viremia prior to ICU admission further complicates interpretation, as the onset and duration of viremia before study inclusion remain unknown, limiting assessment of the complete viral trajectory. Furthermore, CMV serostatus was not available for all patients, preventing definitive differentiation between CMV reactivation and primary infection. Finally, the analysis does not provide sufficient data to determine whether initiation or non-initiation of antiviral therapy influenced the development of clinically manifest CMV disease, but only serves as a hypothesis-generating study. Taken together, our findings show that among critically ill patients with CMV viremia three distinct clusters can be distinguished. Patients with markedly elevated initial viral loads had the poorest survival and highest rates of probable CMV pneumonia. Nearly all received antiviral therapy, reflecting clinician concern. The two clusters with moderate viremia displayed divergent viral kinetics (increasing vs. stable), yet outcomes were similar. Antiviral therapy was inconsistently used, and no differences were observed in of the short-term viral kinetics. These findings emphasize that the CMV–DNA threshold for treatment initiation in ICU patients as well as the effect of the treatment itself is currently uncertain, supporting the hypothesis that viral burden may primarily reflect disease severity rather than represent a modifiable causal driver. This study can be used as foundation for future prospective studies with predefined sampling protocols and independent validation cohorts to further explore the prognostic and treatment-related significance of the identified viral trajectories.

## Supplementary Information

Supplementary Material 1.

## Data Availability

The data sets used and/or analyzed during the current study are available from the corresponding author on reasonable request.
