# Supplementary material for: Time-series clustering analysis reveals distinct patterns of cytomegalovirus viremia in critically ill adults
Source: Intensive Care Med Exp. 2026 Feb 12;14:16. doi: 10.1186/s40635-026-00866-9 (PMC12901805; doi:10.1186/s40635-026-00866-9)

**Supplements**

**Supplement figure 1**: Clustered longitudinal viral load trajectories of patients with CMV viremia $\geq$ 1000 IU/mL on the intensive care unit.


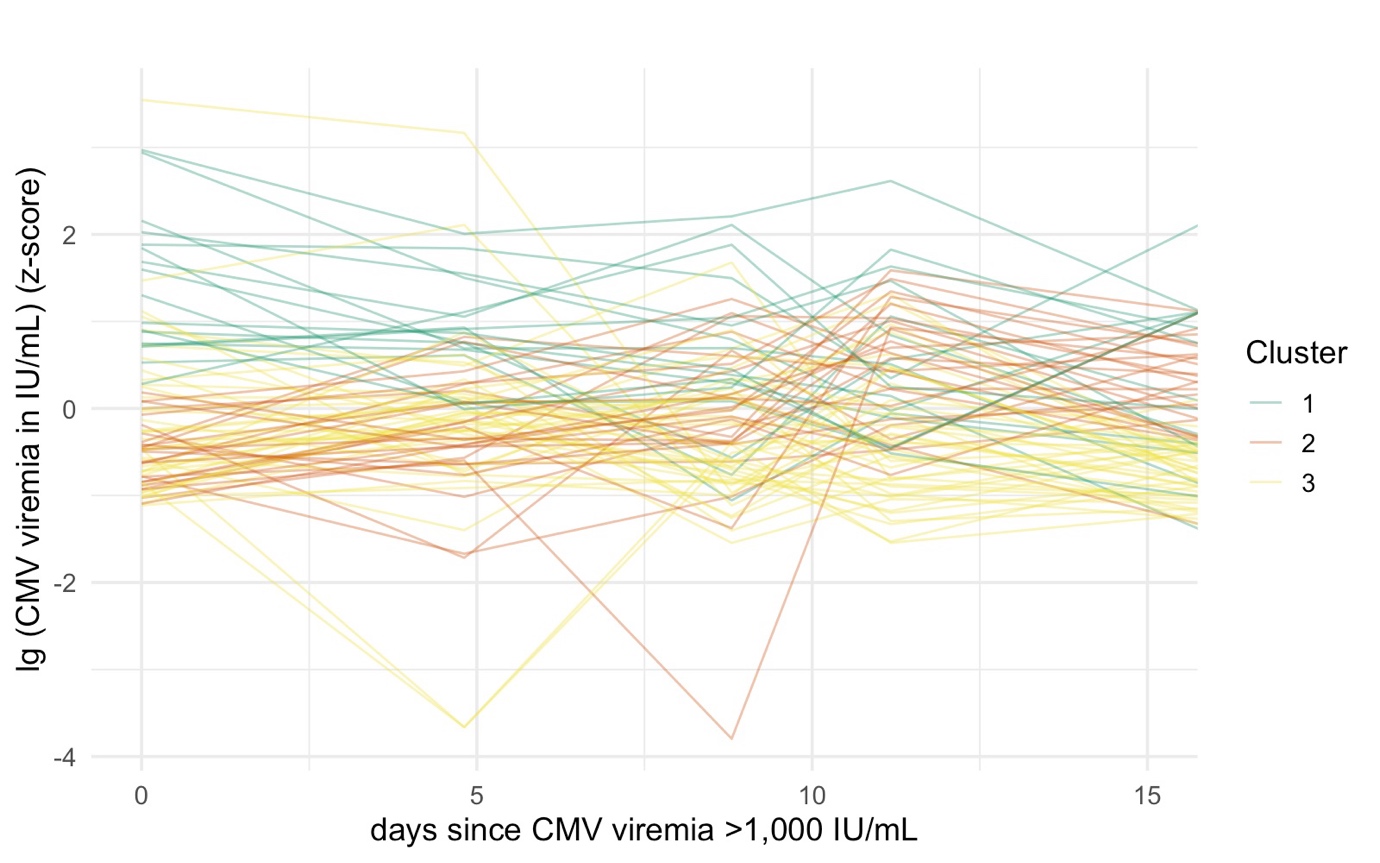


**Supplement table 1:** Patient characteristics of patients with CMV viremia $\geq$ 1000 IU/mL on the intensive care unit with and without CMV-directed antiviral treatment (treatment initiation $\pm$3 days of viremia).

| **Demographics** | **N** | **No treatment** N = 43*^1^* | **Treatment** N = 70*^1^* | **p-value***^2^* |
| --- | --- | --- | --- | --- |
| age | 113 | 60.0 (49.0, 69.0) | 59.5 (46.0, 67.0) | 0.6 |
| sex | 113 |  |  | 0.038 |
| female |  | 17 (40%) | 15 (21%) |  |
| male |  | 26 (60%) | 55 (79%) |  |
| Immunosuppression | 113 | 30 (70%) | 55 (79%) | 0.3 |
| HIV | 113 | 6 (14%) | 10 (14%) | >0.9 |
| SOFA score* | 113 | 7.0 (5.0, 10.0) | 6.0 (4.0, 11.0) | 0.7 |
| APACHE score* | 112 | 19.5 (15.0, 23.0) | 21.0 (15.0, 26.0) | 0.5 |
| White blood count* (/nl) | 113 | 9.2 (4.8, 17.6) | 6.2 (1.5, 12.8) | 0.2 |
| Hb* (g/dl) | 113 | 9.5 (7.9, 11.2) | 9.2 (8.1, 10.7) | >0.9 |
| Thrombocytes* (/nl) | 113 | 134.0 (28.0, 271.0) | 109.5 (33.0, 208.0) | 0.3 |
| Creatinine* (mg/dl) | 113 | 1.1 (0.7, 2.1) | 1.0 (0.7, 1.8) | 0.3 |
| Bilirubin* (mg/dl) | 108 | 0.5 (0.4, 1.4) | 0.6 (0.3, 1.4) | 0.9 |
| C-reactive protein* | 113 | 152.8 (57.3, 202.8) | 150.4 (80.1, 192.5) | 0.9 |
| Procalcitonin | 89 | 0.6 (0.2, 4.6) | 1.0 (0.3, 3.3) | 0.4 |
| Lactate | 104 | 1.7 (1.2, 2.6) | 1.6 (1.2, 3.1) | 0.8 |
| LDH | 111 | 430 (281, 600) | 392 (276, 645) | 0.6 |
| CMV copies at reactivation (IU/mL) | 113 | 1,930 (1,450, 3,330) | 7,370 (2,770, 28,300) | <0.001 |
| maximum CMV copies (IU/mL) | 113 | 2,350 (1,556, 5,030) | 6,020 (2,620, 24,700) | <0.001 |
| proven CMV gastrointestinal disease | 113 | 2 (4.7%) | 11 (16%) | 0.13 |
| probable CMV pneumonia | 113 | 3 (7.0%) | 20 (29%) | 0.006 |
| *^1^* Median (Q1, Q3); n (%);*^2^* Wilcoxon rank sum test; Pearson’s Chi-squared test; Fisher’s exact test | | | | |
| * at admission | | | | |

**Supplement table 2:** Treatment and outcome of patients with CMV viremia $\geq$ 1000 IU/mL on the intensive care unit with and without CMV-directed antiviral treatment (treatment initiation $\pm$3 days of viremia).

| **Treatment & Outcome** | **N** | **No treatment**  N = 43*^1^* | **Treatment** N = 70*^1^* | **p-value***^2^* |
| --- | --- | --- | --- | --- |
| CMV prophylaxis | 113 | 1 (2.3%) | 3 (4.3%) | >0.9 |
| antibiotics | 113 | 36 (84%) | 68 (97%) | 0.026 |
| antimycotics | 113 | 10 (23%) | 24 (34%) | 0.2 |
| antiretroviral therapy | 113 | 2 (4.7%) | 8 (11%) | 0.3 |
| vasopressors | 113 | 27 (63%) | 54 (77%) | 0.10 |
| mechanical ventilation | 113 | 25 (58%) | 48 (69%) | 0.3 |
| high flow oxygen therapy | 113 | 12 (28%) | 31 (44%) | 0.082 |
| non-invasive ventilation | 113 | 15 (35%) | 26 (37%) | 0.8 |
| ECMO | 113 | 3 (7.0%) | 5 (7.1%) | >0.9 |
| acute renal injury | 113 | 20 (47%) | 33 (47%) | >0.9 |
| dialysis | 113 | 17 (40%) | 21 (30%) | 0.3 |
| Cardiopulmonary resuscitation | 113 | 8 (19%) | 16 (23%) | 0.6 |
| ICU length of stay | 113 | 15.0 (7.0, 38.0) | 19.5 (10.0, 34.0) | 0.6 |
| ICU survival | 113 | 23 (53%) | 33 (47%) | 0.5 |
| hospital survival | 113 | 20 (47%) | 27 (39%) | 0.4 |
| 30-day survival | 113 | 24 (56%) | 37 (53%) | 0.8 |
| 60-day survival | 103 | 19 (46%) | 21 (34%) | 0.2 |
| 1 year survival | 91 | 12 (32%) | 9 (17%) | 0.10 |
| *^1^* n (%); Median (Q1, Q3) | | | | |
| *^2^* Fisher’s exact test; Pearson’s Chi-squared test; Wilcoxon rank sum test | | | | |

**Supplement figure 2:** Kaplan-meier analysis of patients with CMV viremia $\geq$ 1000 IU/mL on the intensive care unit, subdivided by CMV-directed antiviral treatment.


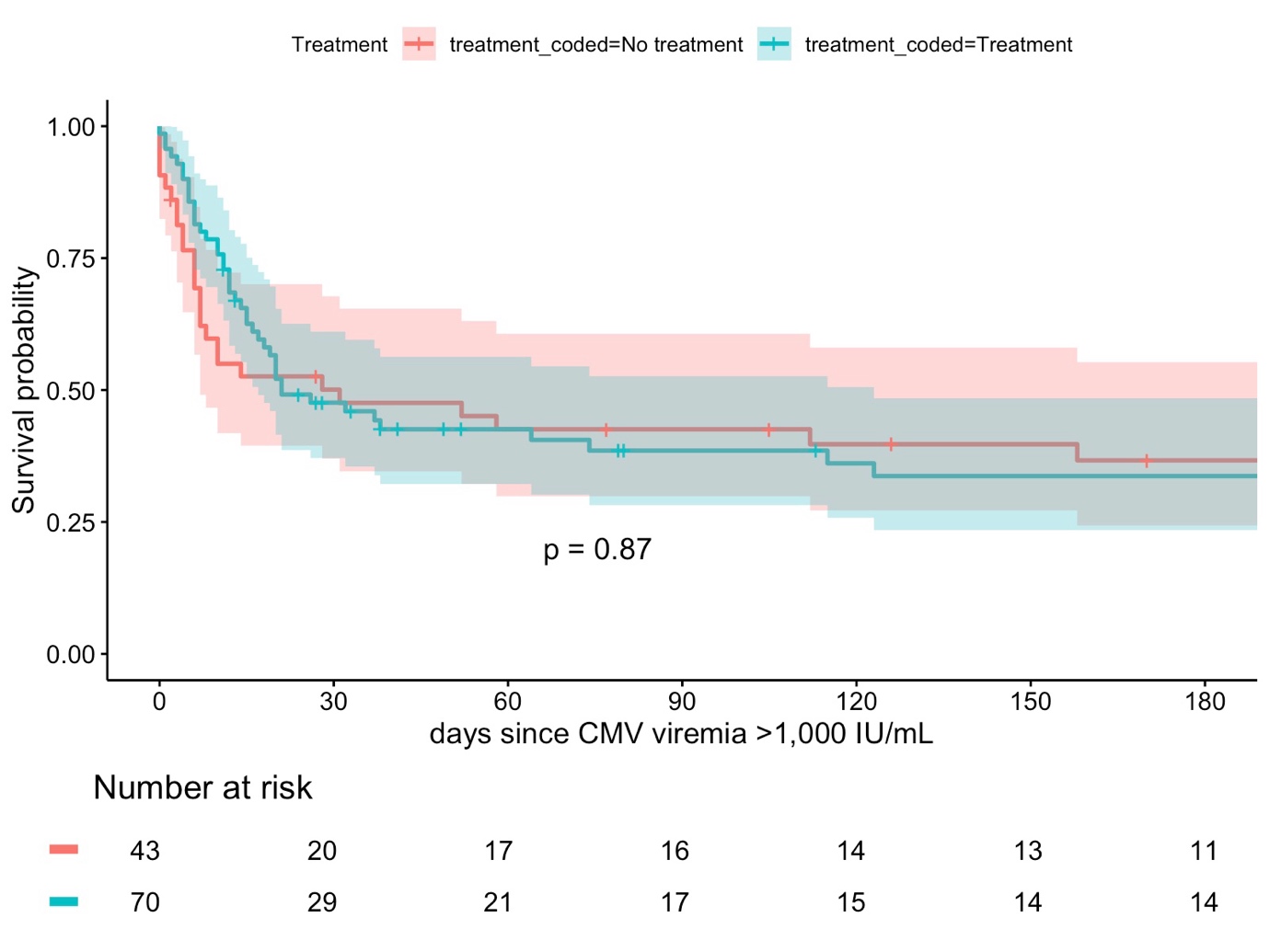

Supplement: Supplementary file 1 — Supplementary Material 1. [file 40635_2026_866_MOESM1_ESM.docx]
